# HKT Transporters—State of the Art

**DOI:** 10.3390/ijms141020359

**Published:** 2013-10-14

**Authors:** Pedro Almeida, Diana Katschnig, Albertus H. de Boer

**Affiliations:** 1 Vrije Universiteit Amsterdam, Faculty Earth and Life Sciences, Department of Structural Biology, Amsterdam NL-1081 HV, The Netherlands; E-Mail: a.h.de.boer@vu.nl; 2 Vrije Universiteit Amsterdam, Faculty Earth and Life Sciences, Systems Ecology, Department of Ecological Science, Amsterdam NL-1081 HV, The Netherlands; E-Mail: d.katschnig@vu.nl

**Keywords:** abiotic stress, salinity stress, HKT transporters

## Abstract

The increase in soil salinity poses a serious threat to agricultural yields. Under salinity stress, several Na^+^ transporters play an essential role in Na^+^ tolerance in plants. Amongst all Na^+^ transporters, HKT has been shown to have a crucial role in both mono and dicotyledonous plants in the tolerance to salinity stress. Here we present an overview of the physiological role of HKT transporters in plant Na^+^ homeostasis. HKT regulation and amino acids important to the correct function of HKT transporters are reviewed. The functions of the most recently characterized HKT members from both HKT1 and HKT2 subfamilies are also discussed. Topics that still need to be studied in future research (e.g., HKT regulation) as well as research suggestions (e.g., generation of HKT mutants) are addressed.

## 1. Introduction

Amongst abiotic stresses, soil salinity is one of the major factors affecting agriculture and plant productivity [1]. Salinization is urgently becoming more serious as the area of land affected by salinity is constantly increasing due to climate change or irrigation with poor quality water [2,3]. In order to fight the problem of salinity, a better understanding of the physiological mechanisms involved in ion homeostasis in the plant needs to be achieved. Amongst the salts that accumulate in soils NaCl is most prominent. Inside the plant, Na^+^ has detrimental effects on cell functioning, by interfering negatively with protein and membrane stability and causing ROS production. In order to control Na^+^ homeostasis, plants have different Na^+^ transporters to protect the plant against damage due to Na^+^ accumulation: antiporters in the root that extrude Na^+^ back to the soil in a mechanism coupled to H^+^ transport (involving the SOS pathway) [4,5]; transporters that retrieve Na^+^ from the transpiration stream avoiding the over-accumulation of Na^+^ in the photosynthetic tissues (involving HKT transporters) [6,7]; and antiporters that sequester Na^+^ in the vacuoles (involving NHX1 antiporters), along the electrochemical gradient created by the H^+^-ATPase and the H^+^-PPase [8]. HKT transporters, with special emphasis on members of class I, are one of the most studied Na^+^ permeable transporters [9]. These Na^+^ transporters, often located in xylem parenchyma cells and root epidermal cells have been found in many plant species and recent studies have shown their crucial importance in salinity tolerance in both mono- and dicotyledonous species [10–13]. This makes HKT transporters a preferential target for the engineering of plant stress tolerance.

This review will discuss the latest research developments on HKT transporters in mono- and dicotyledonous plants paying attention to the recently characterized HKT members, data on ion selectivity, HKT regulation and residues in the HKT protein with important functions for the transport characteristics of the respective proteins.

## 2. HKT1 *vs*. HKT2—Does the Nomenclature still Hold?

Since the discovery of TaHKT2;1 in 1994 [14], many more HKT transporters from other species and with different transport characteristics have been discovered, eliciting a lively debate about the *in planta* role of these transporters. Upon discovery, HKT transporters from various species received the same name independent from their different transport characteristics [14,15]. In 2006 an international agreement was reached on HKT nomenclature with a classification in two groups according to their transport characteristics [16], with differences in the amino acid in the first pore domain (PD) of the protein as the main distinguishing feature [17,18]. Members of class I (Table 1) possess a serine (S) at this position (the other 3 PD’s have a glycine (G) residue forming a motif of S-G-G-G), whereas members of class II (Table 2) possess a G in all PD’s (G-G-G-G motif) [16]. The presence of either an S or G at this particular position determines the selectivity of the transporter [17]. The presence of an S is associated with a preference for Na^+^ conductance over that of other cations, whereas the presence of a G enables the transporters to select for either Na^+^ and/or K^+^ depending on the external concentrations of these two ions [11]. The role of the S and G in the first PD is explained in more detail in the Section: *Residues important in the correct HKT functioning*.

Although the simplicity of this classification makes it appealing, reality shows that the list with exceptions is growing. A larger number of exceptions to this list might bring again confusion over the nomenclature and transport characteristics, as the selectivity and affinity of many HKT transporters are different from that indicated by their name. From all the exceptions, OsHKT2;1 is probably the most studied transporter [1,11,22,23,30,33]. Although OsHKT2;1 possesses an S in the first PD it can transport K^+^[11,31]. Another rice HKT transporter with an unusual conductance is OsHKT2;4 [34–36]. OsHKT2;4 is able to transport both Ca^2+^ and Mg^2+^[34,35]. Another exception to the rule is TsHKT1;2, which has an S in the first PD, but has a higher affinity for K^+^ than Na^+^[46]. Surprisingly, this seems to be due to other amino acid residues in the protein than those present at the first PD.

## 3. Class I HKT Transporters—Essential Roles in Na^+^ Detoxification

As discussed above, Class I HKT transporters are low affinity transporters with specificity for Na^+^[3]. Some of these members locate to the plasma membrane of root stele cells, in particular, xylem parenchyma cells (XPC). They function by retrieving Na^+^ from the xylem sap and prevent Na^+^ from reaching the shoots and damage photosynthetic cells. The number of HKT1 family members varies between mono- and dicotyledonous plants. Monocotyledonous plants have more HKT1 members than dicotyledonous [1,10,18,22]. All class I HKT1 transporters isolated from monocotyledonous plants and characterized so far show selectivity for Na^+^ only [1,10,18,22]. In this section we will discuss the roles of several Class I HKT transporters with emphasis on AtHKT1;1, OsHKT1;4/5 and TaHKT1;4/5 and their roles in *Arabidopsis*, rice and wheat.

### 3.1. Arabidopsis AtHKT1;1

The first Class I HKT1 member was *is*olated from *Arabidopsis thaliana* [15]. When expressed in *Xenopus laevis* oocytes AtHKT1;1 showed Na^+^ selective transport activity independent from K^+^[15]. Mutant K^+^ uptake yeast cells (*Δtrk1 Δtrk2*) transformed with *AtHKT1;1* were not able to grow on medium containing 1 mM K^+^ and their growth was reduced in medium containing Na^+^[15].

At the plant level, AtHKT1;1 was first suggested to mediate Na^+^ uptake from the external medium [47]. This AtHKT1;1 function was soon discarded as experiments done with *Arabidopsis* wild-type and *athkt1;1* mutant plants revealed no differences in root Na^+^ uptake [19,48,49]. Besides, *AtHKT1;1* is expressed in the root stele and in the leaf vasculature but not in the root tips, which would not favour a role in Na^+^ uptake from the external medium [6]. *AtHKT1;1* expression was shown in phloem cells of both roots and leaves [19]. Later, immunological detection of AtHKT1;1 in cross-sections of vascular bundles of *Arabidopsis* leaves showed localization of AtHKT1;1 to the plasma membrane of XPC [21]. Cell-specific *AtHKT1;1* expression in the pericycle [20] and in the stelar [12] cells of *Arabidopsis thaliana* plants via an enhancer trap system [50] resulted in decreased Na^+^ accumulation in the shoots and an increased NaCl tolerance in comparison to the parental lines [12,20]. A similar experiment where *AtHKT1;1* was expressed in the root cortex of rice showed the same increase in Na^+^ sequestration in the roots and reduced transport to the shoots with an increase in salinity tolerance of transgenic rice plants [20]. These results suggest, that in *Arabidopsis*, over-expression of *AtHKT1;1* in the roots, independently of the tissue, results in improved salinity tolerance through higher Na^+^ sequestration of Na^+^ in the root and reduced Na^+^ transport to the shoots. Interestingly, over-expression of *AtHKT1;1* under the CaM-^35^S promoter [12] or even the endogenous promoter [51] rendered the plants more salt sensitive, possibly due to an increased unidirectional influx of Na^+^ into the roots of these plants [12]. The results on the *AtHKT1;1* cell specific expression [12,20] indicate that the improvement of salinity tolerance requires cell type-specific *AtHKT1;1* over-expression.

All results obtained with AtHKT1;1 show that the main role of AtHKT1;1 *in planta* is to avoid the accumulation of excessive Na^+^ in the shoots of *Arabidopsis* [12,19–21]. Na^+^ exclusion from the shoots and photosynthetic tissues has long been proposed as a mechanism of salinity tolerance [7,10,12,20,25,45,52–54]. However, two *Arabidopsis* ecotypes collected from the coastal areas of Tossa del Mar, Spain and Tsu, Japan, (Ts-1 and Tsu-1), respectively, accumulate higher shoot levels of Na^+^ than Col-0 due to the presence of a weak *AtHKT1;1* allele that is not expressed in the roots [55]. The lack of correlation between shoot Na^+^ concentration and salinity tolerance in *Arabidopsis thaliana* ecotypes has been described as well [56]. In contrast with *athkt1;1* mutant plants, this novel *AtHKT1;1* allele in Ts-1 and Tsu-1does not confer NaCl sensitivity, and, in fact, co-segregates with higher tolerance to NaCl [55]. The analyses of the offspring produced by a cross between Tsu-1 and Col-0 wild-type revealed that Tsu-1 *HKT1;1* homo- and heterozygous plants were able to survive longer in the presence of 100 mM NaCl in comparison to Col-0 homozygous plants [55]. Another explanation for this phenotype is, that a second unknown gene and not the weak *AtHKT1;1* allele is responsible for the higher salinity tolerance. Due to a small genetic distance between this unknown gene and *AtHKT1;1*, both segregate together [55]. Although a great effort has been made, more research is necessary to unravel the role of AtHKT1;1 unequivocally in the shoots of *Arabidopsis thaliana.*

### 3.2. Rice OsHKT1;x

In contrast to the small number of HKT1 members present in dicotyledonous plants, monocotyledonous plants have several HKT1 genes [7,10,13,18,22,24,25]. In monocotyledonous plants the physiological role of HKT1 members *in planta* was revealed through the discovery that a QTL controlling the accumulation of Na^+^ in the leaves of rice and wheat was traced down to Class I HKT transporters [7,10,25]. In rice, a trait (*SKC1*) involved in maintaining higher shoot K^+^ concentrations during salinity stress was identified, isolated and characterized [10]. Comparison of *SKC1* cDNA with available database sequences showed that *SKC1* corresponded to *OsHKT1;5* (*OsHKT8*) [10]. Analysis of gene expression, Na^+^ and K^+^ accumulation in the roots and in the shoots and heterologous expression in *Xenopus laevis* oocytes further supported a similar role of OsHKT1;5 in rice [10] as for AtHKT1;1 in *Arabidopsis thaliana* [15,21]. Rice contains four more HKT1 members in its genome, *OsHKT1;1*, *OsHKT1;2*, *OsHKT1;3* and *OsHKT1;4* [10,18,22,24].

In saline conditions, *OsHKT1;4* is expressed in the sheaths [24]. Although three different *OsHKT1;4* transcripts are present in the rice lines Pokkali and Nipponbare, only the amount of the correct spliced transcript inversely correlates with the concentration of Na^+^ in individual leaf blades in both lines [24]. This points clearly to the role of the full length OsHKT1;4 in control of the sheath-to-blade transfer of Na^+^ in the shoots of rice [24].

*OsHKT1;1* is expressed in the roots (epidermis, exodermis and cortex differentiated into aerenchyma) and in the shoots it is expressed in bulliform cells and vascular tissues, both xylem and phloem [18,22]. When expressed in yeast cells, only Na^+^ transport is observed and kinetic analysis showed a low affinity Na^+^ uptake competitively inhibited by K^+^ and Rb^+^, although Rb^+^ was not transported by OsHKT1;1 [18]. In *Xenopus oocytes* OsHKT1;1 shows characteristics of a Na^+^ inward rectifying transporter mediating no outward currents; the currents display voltage-dependent properties upon hyperpolarized pulses [22]. The expression of *OsHKT1;1* in bulliform cells might point to different physiological roles *in planta* in comparison to other rice HKTs.

Although mainly expressed in the shoots, *OsHKT1;3* is also detected in the roots, the cortex and in the vascular tissues of the stele. In comparison to *OsHKT1;1* and *OsHKT2;1* it shows a stronger labelling in the phloem [22]. In the leaves, it is expressed in bulliform cells where the staining is particularly strong, but also in the vascular tissues, both xylem and phloem [22]. Although yeast cells expressing *OsHKT1;3* did not mediate any type of transport [18], in *Xenopus laevis* oocytes OsHKT1;3 mediated both inward and outward Na^+^ currents with weak inward rectification [22]. OsHKT1;2 has not been thoroughly studied as other HKT members in rice. *In planta*, OsHKT1;2 was not detected in the roots and in the leaves its expression did not change significantly upon treatment with Na^+^ and K^+^[23].

### 3.3. Wheat TaHKT1;4/5

In wheat, QTL analyses using a novel durum wheat, Line 149, (*Triticum turgidum* L. subsp. *Durum*), identified two loci, *Nax1* [43], and *Nax2* [7], involved in Na^+^ exclusion from the xylem and reduced Na^+^ export to the shoots [7,43]. Moreover, the hexaploid bread wheat (*Triticum aestivum*) containing the genomes A, B and D, is more salt tolerant than the tetraploid durum wheat containing the genomes A and B [57,58]. It was discovered that the D genome carries a locus (*Kna1*) responsible for maintenance of high K^+^/Na^+^ ratio during salt stress which renders bread wheat salt tolerant [58,59]. The three *Triticum* sp QTLs *Nax1*, *Nax2* and *Kna1* control both the removal of Na^+^ from the xylem and the accumulation of Na^+^ in leaf sheaths [7,25,60]. Using fine mapping *Nax1* and *Nax2* were identified as members of the *HKT1;4* gene family [25] and *Kna1* as member of the *HKT1;5* gene family [7]. Because both *Nax* genes originated from a wheat relative, *Triticum monococcum*, that was crossed with a durum wheat, they were named *TmHKT1;4-A2* and *TmHKT1;5-A*, respectively [60,61]. The *Nax2* region of Line 149 was found to correspond to the *Kna1* region of the bread wheat and *Kna1* was named *TaHKT1;5-D* [7]. These genes clearly have similar functions as *AtHKT1;1* in *Arabidopsis* and *OsHKT1;5* and *OsHKT1;4* in rice [10,21,24,61,62], what was supported by the reduction of the Na^+^ accumulation in the leaves of bread wheat plants, where both *Nax1* and *Nax2* genes were introduced through conventional hybridization, growing under saline and water logged conditions [60]. Moreover, field trials in saline soils done with durum wheat carrying the *TmHKT1;5-A* gene showed that leaf Na^+^ accumulation was reduced and grain yield increased by 25% compared to near-isogenic lines without the *Nax2* locus [13]. These results showed that *HKT* genes have a crucial role in the salinity tolerance of wheat plants.

### 3.4. Tomato HKT1;1 and HKT1;2

In tomato (*Solanum lycopersicum)* two *HKT* genes were detected to be closely linked [26]. These two genes were analysed by heterologous expression in a yeast strain mutated for K^+^ uptake. Both SlHKT1;1 and SlHKT1;2 were unable to complement the growth of the yeast mutant in the presence of low K^+^ in the medium. Cells expressing *SlHKT1;1* were able to deplete external Na^+^, showing that SlHKT1;1 is a Na^+^-selective transporter [26]. No transport activity was detected in yeast cells expressing *SlHKT1;2* for either Na^+^ or K^+^[26]. The Na^+^ selectivity of SlHKT1;1 and the absence of transport activity in SlHKT1;2 were also observed in our lab in experiments where *SlHKT1;1* and *SlHKT1;2* expressing oocytes were characterized (Almeida and de Boer, unpublished data). Expression analysis of *SlHKT1;1* and *SlHKT1;2* showed ubiquitous expression in roots, stems, leaves, flowers and fruits. The results by Asins *et al*. [26] suggest that not only in monocots (as done so far), but also in dicot plants, *HKT* genes might be revealed by QTL studies.

## 4. Class II HKT Transporters—A Role for K^+^

In contrast to Class I HKTs, members of Class II HKT have been shown to have a role in Na^+^ uptake from the external medium, particularly when K^+^ is limiting [11,23,37,42]. In K^+^ limiting conditions, HKT2 transporters show an up-regulation in expression [9,11,23,30,33,42].

### 4.1. OsHKT2;1

OsHKT2;1 is an unusual class II transporter [63], as it has an S residue in the first PD and its Na^+^ transport capacity is similar to class I members. However, OsHKT2;1 is also able to transport K^+^ depending on the external concentrations of both Na^+^ and K^+^[9,11,22]. Characterization studies using heterologous expression of *OsHKT2;1* in yeast cells and *Xenopus* oocytes revealed that OsHKT2;1 mediates both inward and outward Na^+^ currents [22]. Depending on the external concentration of Na^+^ and K^+^, OsHKT2;1 showed several permeation modes: Na^+^/K^+^ symport when Na^+^ and K^+^ were present at sub-millimolar concentrations, Na^+^ uniport in the presence of Na^+^ at millimolar range and K^+^ at sub-millimolar range, or inhibited states when K^+^ was present in the millimolar range up to 10 mM [18,22,30]. In roots, *OsHKT2;1* is expressed in peripheral layers (epidermis, exodermis and cortex differentiated into aerenchyma), which agrees with a function in ion uptake from the external medium. In the stele it is mainly expressed in the phloem and in the leaves it is expressed in bulliform cells and vascular tissues, both xylem and phloem [22]. *In planta*, OsHKT2;1 takes up Na^+^ from the external medium as concluded from the large reduction in Na^+^ uptake observed in the *oshkt2;1* mutant in comparison to wild type plants [11].

The K^+^ starvation driven Na^+^ uptake mediated by OsHKT2;1 in rice is the so-called nutritional Na^+^ absorption [11] that is used by plants when grown at high salinity conditions and that allows for the replacement of K^+^ by Na^+^[11,64]. In these conditions moderate levels of Na^+^ are beneficial as they can be used in osmotic balance [65]. The replacement of K^+^ by Na^+^ is, however, limited as high external concentrations of Na^+^ down-regulate *HKT*, especially *OsHKT2;1* [11,18]. Although in rice only OsHKT2;1 is known to be involved in nutritional Na^+^ absorption, accumulation of *mRNAs* of other rice HKT members were also reduced by external Na^+^ concentrations of 30 mM [30].

### 4.2. TaHKT2;1

In wheat, TaHKT2;1 seems to have a similar function in root Na^+^ influx as OsHKT2;1 has in rice [9]. *TaHKT2;1* is expressed in the root cortex [14], and like *OsHKT2;1* in rice, *TaHKT2;1* expression is enhanced by K^+^ starvation [66]. In cortex cells, K^+^ starvation enhanced Na^+^ influx currents [67]. When expressed in the salt sensitive G19 yeast cells, an increased Na^+^ sensitivity of the cells was observed [38,39], although not as strong as the sensitivity caused by OsHKT2;1 [30]. When expressed and analysed in *Xenopus laevis* oocytes, TaHKT2;1 mediated both Na^+^ and K^+^ transport [14], and probably Mg^2+^[34]. *In planta*, a role of TaHKT2;1 in K^+^ uptake seems unlikely [68] and a role in Mg^2+^ uptake was not tested. The anti-sense repression of *TaHKT2;1* expression in wheat plants resulted in a decrease in Na^+^ uptake by the roots and reduced Na^+^ translocation to the shoots, but K^+^ homeostasis was not affected [37]. These results provide evidence that TaHKT2;1 most probably, has a role in Na^+^ transport [37,42]. The results of the repression of *TaHKT2;1* expression support the notion that Na^+^ exclusion from the shoots is an essential mechanism in wheat salinity tolerance [37].

### 4.3. HvHKT2;1

Like other HKT2;1 members of monocots, *HvHKT2;1* is preferentially expressed in the root cortex and to a much lower level in leaf blades and sheaths. Its expression is up-regulated by low external K^+^ and high Na^+^ in both roots and shoots, and in shoots, respectively [42]. When expressed in both yeast cells and *Xenopus laevis* oocytes HvHKT2;1 is shown to co-transport Na^+^ and K^+^[41–43]. In oocytes, like OsHKT2;1, the transport characteristics vary according to the external concentrations of Na^+^ and K^+^.

HvHKT2;1 differs from TaHKT2;1 in the capacity to maintain the K^+^ transport activity in the absence of Na^+^. TaHKT2;1 requires the presence of Na^+^ to be able to take up K^+^, whereas the K^+^ uptake by HvHKT2;1 is maintained in the absence of Na^+^, although it is reduced [42]. Transgenic barley lines over-expressing *HvHKT2;1* showed higher growth rates in the presence of both 50 and 100 mM Na^+^ and a constant K^+^ concentration of 2 mM. Interestingly, the over-expressing barley plants displayed higher Na^+^ concentrations in the xylem, enhanced translocation of Na^+^ to the shoots and higher Na^+^ accumulation in the leaves than the wild type control plants. Over-expression of *HvHKT2;1* reinforced the includer phenotype of barley resulting in more salt tolerant plants [42]. The over-expression of *HvHKT2;1* also resulted in increased K^+^ contents in plants grown in the “absence” of K^+^ suggesting that HvHKT2;1 could be involved in the root K^+^ absorption at very low concentrations of K^+^[42].

In conclusion, these three HKT2;1 transporters display common properties in their cell specific expression and their ability to transport both Na^+^ and K^+^ when expressed in heterologous systems. They also show inhibition of Na^+^ transport by K^+^, what becomes visible above a certain threshold of Na^+^ concentration [14,30,42].

## 5. Other Class II HKT Members

### 5.1. OsHKT2;2

The salt tolerant Nona Boktra and Pokkali cultivars express *OsHKT2;2*, a homologue of *OsHKT2;1*, which is absent in the rice sensitive Nipponbare cultivar [30]. This suggests that the presence of *OsHKT2;2* is an evolutionary advantage for the salt tolerant cultivars [32]. When expressed in *Xenopus* oocytes *OsHKT2;2* shows both Na^+^ transport activity in the presence of K^+^ and *vice versa* [30], and also a small Mg^2+^ permeability [34]. The results for Na^+^ and K^+^ obtained with *Xenopus laevis* oocytes were also observed in *OsHKT2;2* expressing tobacco BY2 cells, showing that OsHKT2;2 works as a Na^+^/K^+^ co-transporter in plant cells as well [33].

### 5.2. OsHKT2;2/1, a New HKT Isoform in Rice

Recently, a chimeric *OsHKT* member from the salt tolerant rice cultivar Nona Bokra was isolated [32]. This member has a 5′ region corresponding to that of *OsHKT2;2*, but a 3′ region corresponding to that of *OsHKT2;1*. This new member was called *OsHKT2;2/1* and it resulted from a 15 kb deletion in chromosome 6 of Nona Bokra, resulting in a chimera between the 5′ end of *OsHKT2;2* and the 3′ end of *OsHKT2;1* [32]. Expression of *OsHKT2;2/1* in *Xenopus laevis* oocytes or *Saccharomyces cerevisiae* cells revealed that OsHKT2;2/1 is permeable to both Na^+^ and K^+^, the latter even in the presence of high external Na^+^ concentrations [32]. This behaviour of OsHKT2;2/1 was more similar to OsHKT2;2 than to OsHKT2;1 [32]. Like *OsHKT2;1* and *OsHKT2;2*, *OsHKT2;2/1* expression in the roots was up-regulated in K^+^ free conditions. In contrast to *OsHKT2;1*, *OsHKT2;2/1* was only expressed in the roots and in conditions of salt stress its expression was reduced but not abolished [32]. Over time, this reduction in *OsHKT2;2/1* expression was less severe than the reduction in *OsHKT2;1* and *OsHKT2;2*, what suggests that OsHKT2;2/1 may play a role in the roots in conditions of low K^+^ concentrations and under salt stress [32].

### 5.3. OsHKT2;3

OsHKT2;3 shows about 93% homology to OsHKT2;4 at the amino acid level [34]. When expressed in the K^+^ uptake-deficient CY162 yeast cells growing under K^+^ limiting conditions, OsHKT2;3 was not able to rescue the K^+^ uptake phenotype. When *OsHKT2;3* was expressed in the salt sensitive G19 yeast cells growing in the presence of NaCl, no differences in growth were observed in comparison to cells transformed with the empty vector [18,34]. Also in *Xenopus laevis* oocytes expressing *OsHKT2;3* no currents were observed in the presence of alkali cations [34]. *OsHKT2;3* is marginally expressed in the roots when compared to the expression in the shoots [18], and its expression does not change due to different Na^+^ and K^+^ concentrations in the growing medium [23]. More research is necessary to unravel the physiological role of this HKT member.

### 5.4. OsHKT2;4, a HKT Member with Unusual Transport Characteristics, Involved in Ca^2+^ Signaling?

OsHKT2;4 is a rice HKT member with unusual transport characteristics, the nature of which still leaves room for discussion [34–36]. When expressed in oocytes, *OsHKT2;4* produced currents containing two components with different kinetics which were different from all HKTs described [35]. It was first reported that, in the presence of Na^+^, K^+^ or even Mg^2+^, *OsHKT2;4* expressing oocytes produced currents that were smaller than those obtained with the mixture of the bath cations including also Ca^2+^[35]. When Ca^2+^ was tested as the current carrier, it generated time dependent currents at hyperpolarizing voltages. Besides Ca^2+^, K^+^ and Na^+^, *OsHKT2;4* expressing oocytes were also permeable to other divalent cations, namely Mg^2+^, Zn^2+^, Mn^2+^, Cu^2+^, Fe^2+^ and Cd^2+^[35]. Based on the results obtained with K^+^ and Ca^2+^ channel blockers, the existence of two binding sites in OsHKT2;4 for cation transport was proposed [35]. However, this hypothesis was refuted by the results of the crystal structure of VpTrkH, a bacterial member of the HKT/Ktr/Trk family [36]. These results confirmed, the presence of one single central pore in each monomeric transporter, not supporting the existence of two distinct permeation pathways [69]. HKT/Ktr/Trk transporters share a typical structure which might have derived from that of an ancestral K^+^ channel subunit [36,70,71].

Later, it was reported that in *OsHKT2;4* expressing oocytes, K^+^ caused the most positive reversal potentials and that both Mg^2+^ and Ca^2+^ had a small permeability that was inhibited by K^+^, suggesting a higher permeability of K^+^ in comparison to the other cations [34]. Increases in the K^+^ concentration in the absence of Na^+^ resulted in shifts of the reversal potential to more positive values showing that OsHKT2;4 transported K^+^ also in the absence of Na^+^ and indicating that OsHKT2;4 was not an obligate Na^+^-K^+^ co-transporter [34]. This implied that OsHKT2;4 was more likely a K^+^ channel/transporter rather than a Na^+^-K^+^ co-transporter [34]. In fact, increases in the external Na^+^ concentration caused only small positive shifts in the reversal potential, showing that OsHKT2;4 transport properties were different from other class II HKT transporters as these showed large positive shifts in the reversal potential when the external Na^+^ concentration was increased [22,28,30,39,72].

Recently, it was reported that *OsHKT2;4* expressing *Xenopus laevis* oocytes did not produce any currents in the presence of either Ca^2+^ or Mg^2+^, even when K^+^ was present at very low concentrations, suggesting that OsHKT2;4 was not permeable to either of these two divalent cations [36]. In this study, the effect of Na^+^ present in excess of K^+^ in the external medium was also analysed. In contrast to the results obtained before [34], Sassi and co-workers reported that in these conditions Na^+^ was the main ion being transported and not K^+^[36]. When K^+^ was present at similar or higher concentrations than Na^+^, the main ion being transported was K^+^, as increments in the Na^+^ concentration were without any effect on the current reversal potential [36]. These last results seem to confirm that, like OsHKT2;1, OsHKT2;4 has also different conduction modes depending on the external Na^+^ concentration [34,36], although OsHKT2;4 shows a high K^+^ and a singularly low Na^+^ permeability as compared with the other class II HKT transporters [22,73].

Although only OsHKT2;2 was shown to transport K^+^ when expressed in BY2 tobacco cells [33], the expression pattern together with the high K^+^ permeability of OsHKT2;4 when expressed in oocytes, might suggest a role of this HKT member in uptake and long distance transport of K^+^*in planta* [34–36].

In conclusion, the presence or absence of OsHKT2;2 and OsHKT2;2/1 seems to be an evolutionary advantage for the salt tolerant cultivars. This advantage might be related to a role in K^+^ homeostasis not only of OsHKT2;2 and OsHKT2;2/1 but also of OsHKT2;4.

## 6. Do HKT Transporters Isolated from Mosses and Clubmosses Form a Third Class?

A Blast search allowed the identification and isolation of an *HKT* member from the moss *Physcomitrella patens* and several *HKT* genes from the club moss *Selaginella moellendorffii* [63]. A phylogenetic tree made with all known HKT protein sequences, revealed that these (club) moss HKT genes do not belong to either of the two HKT subfamilies [16]. A third HKT subfamily was therefore suggested for these (club) moss HKT members because the accepted *HKT* nomenclature [16] cannot be used for these HKT members [63].

The expression of *PpHKT1* in yeast cells defective in K^+^ uptake and unable to take up Na^+^ from low Na^+^ concentrations showed that PpHKT1 mediates both K^+^ and Na^+^ influx [63]. However, when *Physcomitrella patens* wild-type and *pphkt1* mutant moss were grown at different Na^+^ and K^+^ concentrations, including K^+^ starving conditions, no effects were observed on either the growth rate or on the K^+^ or Na^+^ contents. Moreover, the expression of *PpHKT1* remained low and was not affected by either K^+^ starvation, different pH values, or the presence of Na^+^[63]. These results are different from those obtained with both Class I and Class II HKT members characterized so far and might be linked to the different morphology and living conditions of mosses in comparison to mono- and dicotyledonous plants. More research with *HKT* members isolated from other mosses and club mosses is necessary to confirm whether the transport and expression characteristics of *PpHKT1* are similar among mosses and club mosses. It will also be interesting to identify and characterize *HKT* members isolated from plants belonging to old genera like *Gingko* and *Magnoliacea*. This would reveal whether these *HKT* members share more characteristics with primitive or with modern plants.

## 7. HKT Regulation

**Figure 1:**
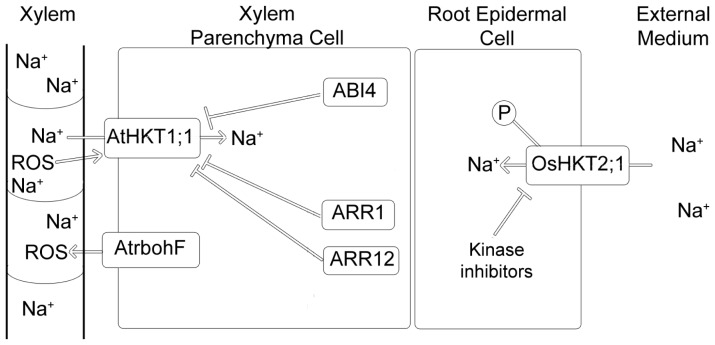
Mechanisms involved in the regulation of HKT transporters. Cytokinins and ABI4 down regulate the expression of *AtHKT1;1* whereas the presence of ROS in the xylem stream affects expression or activity of *AtHKT1;1*. In rice, the application of phosphorylation inhibitors causes a severe reduction in Na^+^ influx in the root epidermal cells.

Although the involvement of HKT transporters in Na^+^ and K^+^ homeostasis (depending on the specific HKT transporter) is well established, the picture of the mechanism(s) that control the expression and activity of HKT transporters is far from complete. Several reports have, however, attempted to bring light to this topic. In this section we will discuss several mechanisms involved in the regulation of *HKT* genes and proteins (Figure 1).

### 7.1. Promoter Structure

Surprisingly little is known about the role of promoter structure in transcriptional regulation of *HKT* genes. Tissue specific regulation of *AtHKT1;1* gene expression seems to be achieved through the action of a distal enhancer element and a small RNA-mediated DNA methylation [74]. The *AtHKT1;1* promoter has two tandem repeats (R1 and R2) that act to repress (R1) or enhance (R2) *AtHKT1;1* expression. Besides, the *AtHKT1;1* promoter also contains a putative small RNA target region that presents higher methylation levels in the leaves in comparison to the roots [74]. It was proposed that this difference in methylation may contribute to the higher *AtHKT1;1* expression in the roots [74]. Both coastal ecotypes *Ts-1* and *Tsu-1* [55], show only one copy of the tandem repeat which is more similar to R1 of Col-0 [74]. This repeat might be associated with the weak *AtHKT1;1* allele present in these two ecotypes and with the higher accumulation of Na^+^ observed in the shoots [55]. This raises an interesting question about the tissue tolerance of Ts-1 and Tsu-1. How does this ecotype combine elevated Na^+^ accumulation in the shoots but also a higher salinity tolerance in comparison to other *Arabidopsis* ecotypes? One explanation is that Ts-1 and Tsu-1 have a better shoot vacuolar Na^+^ sequestration system than Col-0 and use the high Na^+^ levels to reduce their cellular water potential. Another explanation is that, due to the small genetic distance, a second unknown gene responsible for the higher salinity tolerance segregates together with AtHKT1;1 in these two ecotypes [55].

### 7.2. Regulation by ROS

Several studies using *Arabidopsis* mutants lacking detoxification enzymes [75], enzymes involved in the production of ROS [76], or treatment of wild-type plants with inhibitors of enzymes involved in the production of ROS [77], showed that increases and decreases in ROS accumulation *in planta* were related to higher and lower salinity tolerance, respectively. A study with the *Arabidopsis* mutant *atrbohF* (*Arabidopsis thaliana* respiratory burst oxidase protein F), showed that AtRbohF increases root vascular ROS levels in response to salinity, thereby reducing the amount of Na^+^ in the xylem and, consequently, the amount of Na^+^ exported to the shoots [78]. One hypothesis to explain the effect of AtRbohF on xylem Na^+^ levels is that ROS stimulates AtHKT1;1 expression or activity, because AtHKT1;1 is involved in Na^+^ unloading from the xylem (Figure 1) [6,12,19,21,49]. Additionally, it was shown that *AtRbohF* is also expressed in the root vascular tissue with salinity stress enhancing its expression, and that in non-transpiring conditions the *atrbohF* phenotype is not observed [74]. Changes in ROS levels in the vasculature may be an important determinant of transporter activity, since also the activity of the outward rectifying K^+^-channel SKOR (expressed in XPC’s) is enhanced by ROS [79].

### 7.3. Regulation by Cytokinins

It has long been known that salinity stress changes the cytokinin levels in plants [80], and that, in turn, cytokinin plays a role in the response to salt stress [81]. The expression of all cytokinin receptors and several type-A response regulators is affected by salt treatment [82,83] and loss of function mutations in the cytokinin receptor genes make plants less sensitive to salt [83]. A comparison between the *Arabidopsis* type-B regulator double mutant *atarr1-3arr1-12* (from hereon called *atarr1-12*), which makes the plants insensitive to cytokinin, and wild-type plants demonstrated that cytokinin has a role in Na^+^ accumulation in plants. External application of cytokinin resulted in a higher accumulation of Na^+^ in the shoots of wild-type plants (46% increase) as compared to that in the shoots of *atarr1-12* mutant plants (21% increase) in comparison to non-treated controls [81]. The mutant plants are also less sensitive to salt stress [81]. *AtHKT1;1* gene expression analysis showed that the *atarr1-12* mutant has a 6.2 fold higher expression of *AtHKT1;1* in the roots but no significant changes in the shoots in comparison to wild-type, suggesting that *ARR1-3* and *ARR1-12* transcription factors regulate *AtHKT1;1* expression in the roots (Figure 1) [81]. A microarray study also showed that, in the presence of 200 mM NaCl, the *Arabidopsis* cytokinin deficient *ipt1,3,5,7* mutant had a much higher *AtHKT1;1* expression in comparison to the wild type plants [84]. The fact that the expression pattern of *AtHKT1;1* [6,21] and *ARR1-3* and *ARR1-12* [85] overlaps in the vascular tissue of the root further supports the conclusion that cytokinin signalling controls *AtHKT1;1* expression [81]. Also, treatment of wild type plants with external cytokinin resulted, after only 4 hours, in a reduction in *AtHKT1;1* expression by 87%, whereas this same treatment on *atarr1-12* mutants resulted in only a 21% reduction in the *AtHKT1;1* expression, what confirms the role of cytokinin in the regulation of *AtHKT1;1* [81]. Although these results clearly prove the effect of cytokinin on the expression of *AtHKT1;1*, the molecular basis for this regulation is still unknown. It was suggested that the cell specific expression of *AtHKT1;1* could be restricted by ARR1 and ARR12 through induction of repressor genes in specific cell types that reduce *AtHKT1;1* expression [81].

Experiments done with the plant growth promoting rhizobacteria *Bacillus subtillis* strain *GB03* showed that the volatiles released by this bacteria strain induced changes in *AtHKT1;1* expression both in root (reduction) and shoot (increase) [86]. Amongst the volatiles produced, 2,3-butanediol was shown to be the major effector of growth stimulation in the presence of salt [86]. A later study showed that the *Arabidopsis ein2* (*cytokinin/ ethylene-insensitive*) and *cre1* (*cytokinin receptor-deficient*) mutants showed no response to the volatiles produced by *GB03* [87], confirming that the effect of 2,3-butanediol on the *AtHKT1;1* expression is achieved through cytokinin signaling. Whether the GB03 induced reduction in root *AtHKT1;1* expression is mediated by ARR1-3 and ARR1-12 is not known yet.

### 7.4. Regulation by ABI4

ABI4 (*ABSCISIC ACID INSENSITIVE4*) *is* an ABA responsive transcription factor which acts both as activator and repressor of transcription [88]. The *Arabidopsis abi4* mutant displayed enhanced salinity tolerance, whereas *ABI4* over-expressing plants displayed hypersensitivity to salinity, in comparison to wild type plants [89]. This sensitivity was observed from the stage of germination to adult stages. The increased salinity tolerance of *abi4* mutants correlated with higher *AtHKT1;1* expression (Figure 1) in the roots and lower Na^+^ accumulation in the shoots. The opposite was observed for plants over-expressing *ABI4*. The reduced *AtHKT1;1* expression in wild type *Arabidopsis*, in comparison to the *HKT1;1* expression in *abi4* mutant plants, was shown to be due to the binding of ABI4 to the proximal *AtHKT1;1* promoter region. This repressor effect on *AtHKT1;1* expression was absent in *abi4* mutant plants [89]. These results shed more light on the regulation of *AtHKT1;1* expression. Moreover, the fact that the *abi4* mutant showed enhanced salinity tolerance not only at the germination stage but also at later stages of development, suggests that, in the presence of NaCl, *abi4* mutant plants are more adapted to face salinity stress.

## 8. Residues Important for HKT Function

**Figure 2:**
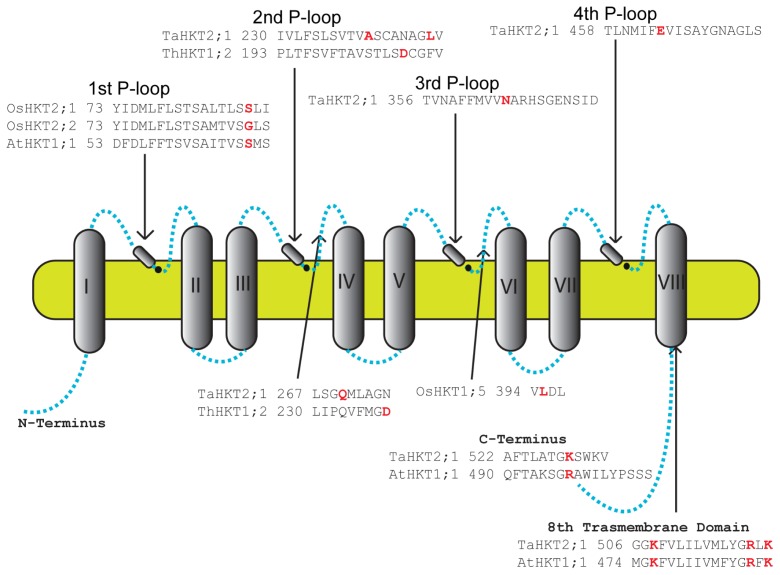
HKT structure and location of specific amino acids that were shown to affect the transport properties when mutated. Alignments show the amino acid sequence of specific domains where these amino acids (highlighted in **red**) are present (different plant species) and have been shown to have a crucial role in the correct functioning of the transporter. The function of the highlighted amino acids has been studied by expression of the mutated proteins in heterologous systems.

As discussed above, certain residues in the HKT transporters have a crucial role in the functioning of the transporter (Figure 2). Here we present the list of all residues that were shown to play an important role in the ion selectivity of several HKT transporters.

*TaHKT2;1*-expressing salt sensitive yeast cells growing in the presence of NaCl, [38,39] allowed the discovery of four *TaHKT2;1* mutants conferring less salt sensitivity to yeast cells. Yeast cells expressing the mutant forms *TaHKT2;1-A_240_V* or *TaHKT2;1-L_247_F* [39], and *TaHKT2;1-Q_270_L* or *TaHKT2;1-N_365_S* [38], showed growth rates higher than those expressing the wild-type transporter. Experiments done with *Xenopus* oocytes confirmed that the four mutations reduced low affinity Na^+^ uptake [38,39]. In *A_240_V-* and *L_247_F-* [39], and in *Q_270_L-* and *N_365_S-* [38] expressing yeast cells the inward low affinity Na^+^ currents were reduced in comparison to wild-type currents. These four mutations reduced the Na^+^ inhibition of high affinity Rb^+^ uptake [38,39], showing the importance of these four residues in the Na^+^ binding capacity of TaHKT2;1. Also in wheat, it was shown that the mutation of glutamate E_464_ to glutamine Q_464_ in TaHKT2;1 affects the normal function of the transporter [90]. K^+^ uptake deficient yeast cells CY162 expressing *TaHKT2;1-E_464_Q* showed enhanced growth in the presence of 50 mM NaCl compared with 0 mM NaCl whereas the growth of *TaHKT2;1* expressing cells was slightly decreased. This enhancement in the growth of TaHKT2;1-E_464_Q was much larger in the presence of low NaCl concentrations (2.5 mM), in contrast to the growth of TaHKT2;1 expressing cells which was reduced at the same concentrations [90]. Na^+^ uptake measurements showed that the E_464_Q mutation reduced the affinity of TaHKT2;1 for Na^+^, by affecting how TaHKT2;1 binds Na^+^ and reducing Na^+^ flux rates but showing no effect on K^+^ binding [90]. These results suggest the involvement of the 4th P-loop region of the TaHKT2;1 in the Na^+^ binding and transport through the transporter.

To study the first PD, several point mutations of the predicted first PD of AtHKT1;1, TaHKT2;1, OsHKT2;1 and OsHKT2;2 proteins were produced and used to study the role of specific amino acids present in the first PD of these HKT transporters. The mutated *AtHKT1;1-S_68_G* and *OsHKT2;1-S_88_G* expressing CY162 yeast cells were able to grow at low concentrations of K^+^ while *AtHKT1;1* and *OsHKT2;2-G_88_S* expressing cells were not [17]. From these experiments it was concluded that the presence of a G at the predicted filter position of the first PD is necessary and sufficient for K^+^ permeation of AtHKT1;1, TaHKT2;1 and OsHKT2;2 [17].

A later paper, where several positive residues from the M_2_D segment of both AtHKT1;1 and TaHKT2;1 were mutated, [91], showed that these residues also have an important role in the normal functioning of HKT transporters in plants. The replacement of arginine (R) R_519_ in TaHKT2;1 and R_487_ in AtHKT1;1 by alanine (A), glutamine (Q), glutamic acid (E) and lysine (K), was analysed in CY162 yeast cells and *Xenopus laevis* oocytes. All yeast cells transformed with *TaHKT2;1* or mutated *TaHKT2;1* were able to grow in the presence of 1 mM KCl, although differences were observed (R_519_K = WT > R_519_Q > R_519_A > R_519_E). Similar experiments done with AtHKT1;1 showed that only oocytes expressing *AtHKT1;1* and *R_487_K* produced Na^+^ currents. These results led to the conclusion that R_519_ in TaHKT2;1 or R_487_ in AtHKT1;1 plays an important role in the ion transport [91]. Other positively charged amino acids present in the M_2_D domain were also mutated namely the lysine (K) K_508_, K_521_, and K_529_ in TaHKT2;1 and K_476_, K_489_, and R_497_ in AtHKT1;1, and the cation transport activity analysed in *Xenopus laevis* oocytes. K_508_Q and R_519_Q showed reduced transport activity compared to K_521_Q and K_529_Q [91]. These results showed that the individual replacement of positively charged amino acids with Q in the M_2_D domain do not delete the cation uptake activity of plant HKT.

*Thellungiella salsuginea*, an *Arabidopsis* relative, possesses two *HKT* genes, *TsHKT1;1* and *TsHKT1;2*, which are induced by NaCl [46]. Because the sodium specific responses of *Arabidopsis* and *Thellungiella* are different with respect to *HKT* expression, *TsHKT-RNAi* lines were created and their salinity sensitivity analysed. *TsHKT-RNAi* lines showed no differences in growth under control conditions but showed sensitivity to sodium compared to the control. RNAi lines growing in hydroponics treated with 250 mM NaCl for 24 h showed decreased K^+^ accumulation and smaller K^+^/Na^+^ ratios in the shoots and marginally higher in the roots pointing to a role of TsHKT1;2 in the maintenance of K^+^ homeostasis [46]. The K^+^ specificity of TsHKT1;2 was found to rely on the presence of two aspartic acid (D) residues located at positions D_207_ and D_238_. When these two residues, individually or together, were replaced by asparagine (N) residues, present in the AtHKT1;1, each single change resulted in the reduction of the growth of the transformed CY162 yeast cells growing in the presence of Na^+^ and low concentrations of K^+^. These results showed that, in the case of TsHKT1;2, which shows a S residue in the first PD, two specific D residues have a strong effect on the selectivity of the transporter [46]. It would be interesting to test whether the mutation of the S present in the first PD into a G has any effect on the K^+^ selectivity of this transporter.

Recently, it was shown that the differences in whole plant Na^+^ (Na^+^ retention in the root and Na^+^ transport rates), between rice cultivars was due to an amino acid substitution in the OsHKT1;5 transporter [24]. Both Pokkali and Nona Bokra cultivars are salt tolerant, presenting a lower total plant Na^+^ accumulation, higher Na^+^ retention in the roots and faster Na^+^ transport rates. These characteristics were associated with the presence of a valine (V) residue at position V_395_ of OsHKT1;5. The salt sensitive Nipponbare cultivar shows, in turn, higher Na^+^ transfer to the shoots and slower Na^+^ transport rates, these being features associated with a leucine (L) residue at position L_395_ of OsHKT1;5 [24]. The V_395_L amino acid substitution is located in close proximity of G_391_ near the entrance of the pore in both transporters [24]. The slower Na^+^ transport rates shown by the OsHKT1;5 of Nipponbare is due to a larger van der Waals volume imposed by the side chain of L_395_[24]. Moreover, the presence of this residue can also influence other residues underlying the pore selectivity within the pore environment [24].

These examples show that besides the residues present at the PD also other residues located throughout the protein have crucial roles in the function of different HKT transporters.

## 9. The Role in Long-Distance Transport

### 9.1. Recirculation *vs.* Exclusion: Evidence for Both Models

Although the ion selectivity of AtHKT1;1 was known [15], the lack of information about the exact cells in which AtHKT1;1 was expressed, did not allow a good understanding of the role of AtHKT1;1 *in planta*. Research done with *athkt1;1* showed that this mutation did ameliorate the *sos3* phenotype and reduced the total amount of Na^+^ in the seedlings, leading to the suggestion that AtHKT1;1 was a root Na^+^ influx pathway [47]. Subsequent studies showed however that the root Na^+^ influx in *athkt1;1* was not lower than in the wild-type *Arabidopsis* plants, discarding the role proposed by Rus and coworkers [19,48]. Nevertheless, immunolocalization of HKT in root tips of *Mesembryanthemum crystallinum*, showed that the protein was most concentrated in epidermal cells which may indicate an important role of McHKT1;1 in cation uptake from the soil [29]. A study performed with EMS *athkt1;1* mutant plants showed that these mutants accumulated less Na^+^ in the phloem sap but not in the xylem sap, when compared to wild-type plants growing in the presence of salinity [19]. This result on phloem Na^+^ content, in addition to a *AtHKT1;1* phloem-specific expression pattern, led to the hypothesis that AtHKT1;1 loads excessive Na^+^ from the shoots into the phloem. In this way, excess Na^+^ in the shoots would be transported back to the roots via the downward phloem flow, the so-called “recirculation” model [19]. Later studies showed that *athkt1;1* mutant plants accumulated Na^+^ to higher levels in the shoots and also in the xylem sap as compared to wild type plants [21]. In this work an AtHKT1;1 antibody and *AtHKT1;1* promoter GUS construct were used to detect the location of the proteins and cell specific expression, respectively. Results from both experiments showed that AtHKT1;1 was present xylem parenchyma cells (XPC) [21]. Based on these results the authors proposed the “exclusion” model [21]. This model proposed that AtHKT1;1 acts by unloading Na^+^ from the xylem sap into XPC in the roots avoiding excessive amounts of Na^+^ to reach the shoots via the transpiration stream [21]. Nowadays the “recirculation” model has been questioned based on the preferential localization of AtHKT1;1 in XPC [12,20,21]. Moreover, data based on unidirectional Na^+^ tracer fluxes indicated a lack of AtHKT1;1 functioning in the recirculation of Na^+^ via the phloem [12,49,53,61]. The xylem Na^+^ unloading function of AtHKT1;1 was also supported by the results of cell specific over-expression of *AtHKT1;1* [12,20]. In these experiments, an enhancer-trap system [50], was used to guide the over-expression of *AtHKT1;1* specifically in the root. The increase in Na^+^ influx into the transformed parenchyma cells created more Na^+^ tolerant plants [12,20].

Although the function of AtHKT1;1 is quite well defined in the roots, the opposite is true for the shoots and the “recirculation” model [19] should not be discarded. In fact, both Na^+^ transport processes could be linked to achieve recirculation of Na^+^ as ions retrieved from the xylem to the XPC could be loaded into the phloem through symplastic diffusion [92]. In cross-sections of *Mesembryanthemum crystallinum* leaves, *McHKT1;1* was detected in vascular bundles and surrounding mesophyll cells [29]. Although the signals in the vasculature were stronger for XPC, also phloem and phloem-associated cells were highlighted [29]. Also in reed plants, PhaHKT2;1 might have a role in Na^+^ recirculation through the phloem [44]. Reed plants of the ecotype Nanpi had a functional PhaHKT2;1 where the ecotype Utsonomiya expressed a splice variant. When treated with salt for 10 days, the plants with the correct splicing variant, Nanpi, contained less Na^+^ in the above ground tissues than Utsonomiya plants and accumulated Na^+^ in the roots. In contrast, Utsonomiya plants had high Na^+^ levels in the shoot [93]. Moreover, in Nanpi plants, the Na^+^ content in the shoots first increased, but decreased to levels in the control plants after 10 days of treatment, while Utsonomiya plants showed a continuous increase upon treatment [93]. These results suggest that in Nanpi plants, the functional HKT2;1 is able to retrieve Na^+^ from the xylem in the roots avoiding high concentrations of Na^+^ from reaching the shoots, but also that in the shoots it might be involved in loading of Na^+^ into the phloem and, consequently, in the recirculation of Na^+^ to the roots. Also in the leaves of two rice varieties, Pokkali and IR29, *OsHKT2;1* was expressed in cells adjacent to phloem vessels suggesting the involvement of this transporter in Na^+^ and K^+^ recirculation [31]. In *Arabidopsis*, although weaker in comparison to the staining in the XPC, GUS staining signals were also found in the shoot in the vicinity of phloem tissues [21]. Thus, the role of HKT transporters in Na^+^ recirculation is not yet clear and more data will be necessary to validate the “recirculation” model [19].

### 9.2. HKT and K^+^ Levels in the Xylem—A Direct or Indirect Effect of HKT

The maintenance of high K^+^ concentrations during events of salinity stress reduces the Na^+^/K^+^ ratio in the plant leaves and is often referred to as crucial for salinity tolerance in glycophyte plants [3]. When first described, AtHKT1;1 was tested in several heterologous systems [15]. Whereas both over-expression in *Xenopus oocytes* and yeast showed AtHKT1;1 to work as a Na^+^ selective uniporter, over-expression in a *E. coli* K^+^ uptake mutant showed an increase in its K^+^ accumulation [15]. *Athkt1;1* plants growing in the presence of 75 mM NaCl had higher concentrations of Na^+^ but lower concentrations of K^+^ present in the xylem and in the shoots [21]. In the rice cultivar Koshihikari, *HKT1;5* encodes a protein with low transport activity which leads to a more salt sensitive phenotype in comparison with the salt tolerant cultivar Nona Bokra [10]. Also here a reduced concentration of K^+^ in the shoot of the salt sensitive rice cultivar was observed when grown in the presence of 140 mM NaCl [10]. In a more recent paper, the over-expression of *AtHKT1;1* in the root stele (through the use of a trap-enhancer system) [50], resulted in the reduction of Na^+^ and in the increase of K^+^ concentration in the shoots of plants growing in the presence of NaCl compared with the controls [12]. In this case it was concluded that this increase in K^+^ shoot concentration was a pleiotropic consequence of the reduced Na^+^ shoot content [12].

As shown by these examples, the mechanisms coordinating the levels of Na^+^ and K^+^ remain obscure, because the literature does not show any evidence for the direct role of AtHKT1;1 in the transport of K^+^*in planta* [12]. Nevertheless, all these observations fit the hypothesis that the uptake of Na^+^ from the xylem into the XPC via HKT1;1 will result in the depolarization of XPCs and activation of K^+^ efflux channels which in turn will release K^+^ into the xylem [21]. This hypothesis also explains the higher K^+^ accumulation in the roots of *athkt1;1* mutant plants [21]. Recently, patch-clamp experiments done with root stelar cells of *AtHKT1;1* wild-type and *athkt1;1* mutant plants showed large currents activated by voltage ramps, in both wild-type and mutant plants, in the presence of 50 mM KCl (bath) and 5 mM KCl (pipette) [94]. Analysis of the reversal potentials and amplitude of the currents formed in both plant types showed no significant differences, suggesting that K^+^ is not substantially transported by AtHKT1;1 [94]. An alternative hypothesis to explain a role for the AtHKT1;1 protein in K^+^-transport is that the AtHKT1;1 protein directly interacts with another cation transport protein and affects the activity of such a protein. The first large-scale interaction screen of membrane and signaling proteins (MIND; http://cas-biodb.cas.unt.edu/project/mind/index.php) shows three putative interaction partners of AtHKT1;1, a glycolyl hydrolase, a glutamate receptor (AtGLR2.9) and KEA3, a cation:proton antiporter. It will be important to find out whether any of these proteins plays a role in K^+^-transport from the root to the shoot.

## 10. Future Prospects

**Figure 3:**
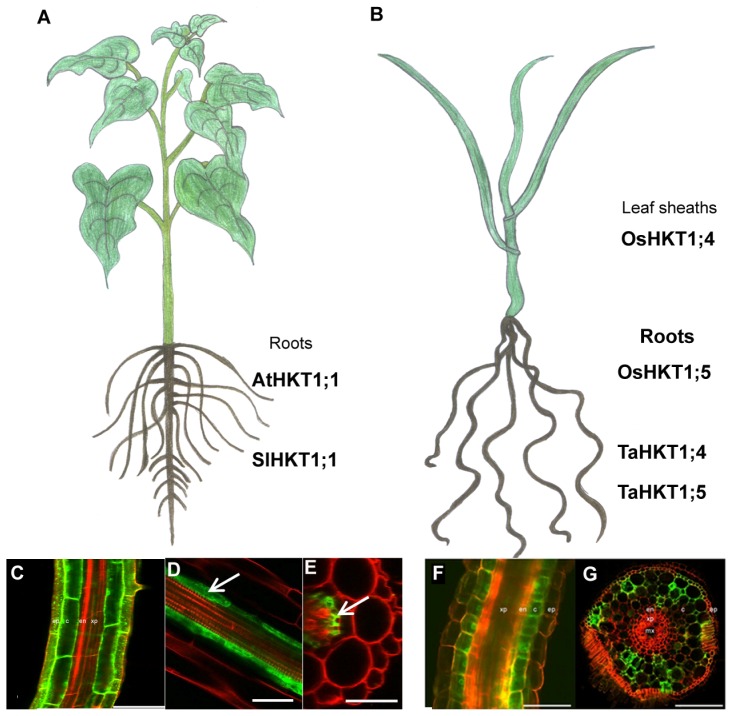
Targeted expression of *AtHKT1;1* in the roots of both (**A**) dicotyledonous and (**B**) monocotyledonous plants. The targeted over-expression of *AtHKT1;1* in the roots, regardless of the tissue as both over-expression in the (**C**) epidermal and cortical cells as well as (**D** and **E**) in the pericycle, resulted in enhanced salinity tolerance in *Arabidopsis thaliana* plants. Also in rice the overexpression of *AtHKT1;1* in the (**F** and **G**) epidermis and cortical cells resulted also in enhanced salinity tolerance. This might be a useful strategy to use with other HKT genes to ameliorate the salt sensitivity of crop species. Bars: (**C**) 75 μm, (**D** and **E**) 40 μm, (**F** and **G**) 100 μm. Figure 3C,F,G reproduced with permission from [20]. Figures 3D,E reproduced with permission from [12].

The constant growth of the world population in combination with the increase in salinized land areas make the generation of more salt tolerant cultivars a goal of utmost importance. Research on the physiological roles of class I HKT transporters like AtHKT1;1, and HKT1;4 and HKT1;5 from rice and wheat, as well as all other discovered HKT transporters will give important information that can be used to engineer salinity tolerant cultivars. For example, the studies by Moller *et al.* and Plett *et al.* provided evidence that targeted over-expression in the roots of both monocotyledonous and dicotyledonous plants results in increased salinity tolerance (Figure 3) [12,20]. As AtHKT1;1, SlHKT1;1 and HKT1;4 and HKT1;5 from both rice and wheat are orthologous and share the same functions *in planta*, this strategy might be useful in the engineering of salt tolerant crop plants.

Although a great deal of information about HKT transporters has been collected in the past years, some questions still need to be addressed and some topics clarified. Are HKT transporters involved in the recirculation of Na^+^ via the phloem, or is this mechanism HKT-independent? Where does the Na^+^ retrieved from the xylem to the XPC go? Also, how is the high Na^+^ mediated down-regulation of class II HKT achieved? With the exception of *oshkt2;1* and *oshkt2;4* [34] mutants, HKT mutants in monocots are still unavailable. The generation of other *HKT* mutants in monocots will be useful to better understand the *in vivo* functions of other HKT transporters already discovered and characterized via heterologous systems. Is the enhanced salinity tolerance revealed by the *Arabidopsis* ecotypes Ts1-1 and Ts-1 related only to the weak allele or also to an unknown gene co-segregating with the *AtHKT1;1* gene? The possibility of another gene involved in salinity tolerance being co-segregated with *AtHKT1;1* in these two ecotypes would allow new approaches to the generation of salt tolerant plants. Are HKT2;1 members involved in high affinity K^+^ uptake *in planta*? Last, should HKT nomenclature be revised? With the increasing number of papers reporting the identification of HKT members with unusual transport characteristics, it will be a matter of time until the actual nomenclature will no longer be able to classify all members in an easy and simple way. And should a third class grouping HKT transporters isolated from mosses and club mosses be formed?

## Figures and Tables

**Table 1:** List of all HKT1 transporters isolated and characterized.

Transporter	Expression in planta	Ref.	Function in planta	Ref.	Transport selectivity when heterologous expressed	Ref.
**AtHKT1;1**	Phloem (roots and shoots)	[19]	Loading excessive shoot Na^+^ into the phloem	[19]	Na^+^ transport (*Xenopus* oocytes)	[15]
	Xylem parenchyma cells	[12,20,21]	Unloading of Na^+^ from the xylem into XPC	[12,20,21]	K^+^ transport (*E. coli*)	
**OsHKT1;1**	In the leaves: bulliform cells and vascular tissues.	[18,22]			Na^+^ transport (*Xenopus* oocytes and *S. cerevisiae* yeast cells)	[18,22]
	In the roots: similar to OsHKT2;1.					
**OsHKT1;2**	Not detected in roots.	[23]				
	Expression does not change with NaCl stress in the leaves.					
**OsHKT1;3**	In the leaves: bulliform cells and vascular tissues, mesophyll cells. In the roots: cortex and vascular tissues in the stele.	[22]			Na^+^ transport (*Xenopus* oocytes)	[22]
**OsHKT1;4**	Leaf sheaths.	[24]	Control of sheath-to-blade transfer of Na^+^	[24]		
**OsHKT1;5**	Mainly expressed in xylem parenchyma cells of both roots and leaves.	[10]	Control of root-to-shoot transfer of Na^+^ by unloading of Na^+^ from the xylem into XPC	[10]	Na^+^ transport (*Xenopus* oocytes)	[10]
**TaHKT1;4**	Expressed in the roots, leaf sheath and leaf blades.	[25]	Unloading of Na^+^ from the xylem into XPC	[25]		
**TaHKT1;5**	Expressed in the roots but not in the shoots.	[7]	Unloading of Na^+^ from the xylem into XPC	[7,13]	Na^+^ transport (*S. cerevisiae* cell)	[13]
					Na^+^ transport (*Xenopus* oocytes)	
					Na^+^ transport (*S. cerevisiae* cells)	[26]
**SlHKT1;1**	Ubiquitously expressed (roots, stems, leaves, flowers, fruits).	[26]			Na^+^ transport (*Xenopus* oocytes)	Almeida *et al.* unpublished results
	NIL and treatment dependent					
**SlHKT1;2**	Ubiquitously expressed (roots, stems, leaves, flowers, fruits). NIL and treatment dependent	[26]			No transport activity detected in either *S. cerevisiae* cells or *Xenopus* oocytes	[26]
						Almeida *et al*. unpublished results
**EcHKT1;1**	Expressed in the leaves, stems and roots	[27]			K^+^ transport (*E. coli* cells)	[27,28]
					Na^+^, K^+^ and Rb^+^ transport (*Xenopus* oocytes)	
**EcHKT1;2**	Expressed in the leaves, stems and roots	[27]			K^+^ transport (*E. coli* cells)	[27,28]
					Na^+^, K^+^, Rb^+^, Li^+^ Transport (*Xenopus* oocytes)	
**McHKT1;1**	In the leaves: xylem parenchyma cells and phloem cells; In the roots: epidermal cells and vascular tissues	[29]	The authors proposed a model where McHKT1;1 Unloads Na^+^ from the xylem in the shoots	[29]	K^+^ transport (*S. cerevisiae* cells)	[29]
					Rb^+^, Cs^+^, K^+^, Na^+^ and Li^+^ transport (*Xenopus* oocytes)	

**Table 2:** List of all HKT2 transporters isolated and characterized.

Transporter	Expression in planta	Ref.	Function in planta	Ref.	Transport selectivity when heterologous expressed	Ref.
**OsHKT2;1**	In the roots: epidermis, exodermis, cortex differentiated into aerenchyma, stele (mainly in the phloem); In the leaves: bulliform cells, xylem, phloem, mesophyll cells	[22]	Nutritional Na^+^ uptake from the external medium	[11]	Na^+^ and K^+^ transport (*Xenopus* oocytes)	[22,30,31]
					Na^+^ transport (*S. cerevisiae* cells)	[18,30]
					K^+^ transport (*S. cerevisiae* cells)	[31]
**OsHKT2;2**	Expressed only in the roots	[32]	Na^+^/K^+^ symporter in BY2 tobacco cells	[33]	Na^+^, K^+^, (*S. cerevisiae* and *Xenopus* oocytes)	[30,34]
			Expected to co-transport both Na^+^ and K^+^ in conditions of K^+^ starvation	[32]		
**OsHKT2;2/1**	Expressed only in the roots	[32]	Expected to co-transport both Na^+^ and K^+^ in the roots in conditions of low K^+^ and under salt stress	[32]	Na^+^ and K^+^ transport (*S. cerevisiae* cells and *Xenopus* oocytes)	[32]
**OsHKT2;3**	Marginally expressed in the roots in comparison to the shoots	[34]			No currents or uptake observed in *Xenopus* oocytes or *S. cerevisiae* cells	[34]
**OsHKT2;4**	Vasculature of primary/ lateral root cells, leaf sheaths, spikelets and the base of stems. Expressed also in mesophyll cells	[35]	Possible role in K^+^ homeostasis as a K^+^ transporter/channel	[34]	Permeable to NH4^+^, Li^+^, Na^+^, K^+^, Ca^2+^, Mg^2+^ Zn^2+^, Mn^2+^, Cu^2+^, Fe^2+^, Cd^2+^ (*Xenopus* oocytes)	[35]
			Possible redundant role in planta as o*shkt2;4* mutants show no phenotype	[35]	Permeable to Na^+^, K^+^, Mg^2+^, Ca^2+^ (*Xenopus* oocytes)	[34]
					K^+^ transport (*S. cerevisiae* cells)	
			Proposed to function as a K^+^ transporter involved in both nutritional K^+^ uptake and long-distance K^+^ transport	[36]	Na^+^ and K^+^ transport (*Xenopus* oocytes)	[36]
**TaHKT2;1**	Root cortical and stele cells Vascular tissue of mesophyll cells	[14]	Na^+^ uptake from the external medium	[37]	Permeable to Na^+^, K^+^, Cs^+^ and Rb^+^ (*Xenopus* oocytes)	[14]
					K^+^ transport (*S. cerevisiae* cells)	
					Na^+^ and K^+^ transport (*S. cerevisiae* cells)	[38,39]
					Na^+^ and K^+^ transport (*Xenopus* oocytes)	
					Permeable to Na^+^, K^+^ and Mg^+^ (*Xenopus* oocytes)	[34]
**PutHKT2;1**	Mainly in roots	[40]	Possible high affinity K^+^ transporter	[40]	Na^+^ and K^+^ transport (*S. cerevisiae* cells)	[40]
**HvHKT2;1**	Root cortex, leaf blades and leaf sheaths	[41,42]	Possible involvement in the root K^+^ (re)absorption at very low K^+^ concentrations	[41,42]	Na^+^ and K^+^ transporter (*S. cerevisiae* and *Xenopus* oocytes)	[41–43]
			Possible uptake of Na^+^ in the roots			
**PhaHKT2;1**	Roots and shoots	[44]			Na^+^ and K^+^ transport (*S. cerevisiae* cells)	[40,44]
**PpHKT1**	-------	-------			Na^+^ and K^+^ uptake (*S. cerevisiae* cells)	[45]
